# EasySMPC: a simple but powerful no-code tool for practical secure multiparty computation

**DOI:** 10.1186/s12859-022-05044-8

**Published:** 2022-12-09

**Authors:** Felix Nikolaus Wirth, Tobias Kussel, Armin Müller, Kay Hamacher, Fabian Prasser

**Affiliations:** 1 Berlin Institute of Health at Charité – Universitätsmedizin Berlin, Medical Informatics Group, Charitéplatz 1, 10117 Berlin, Germany; 2 Computational Biology and Simulation, TU Darmstadt, Darmstadt, Germany

**Keywords:** Secure multi-party computation, SMPC, Secret sharing, GMW protocol, User experience, No-code, Joint calculations

## Abstract

**Background:**

Modern biomedical research is data-driven and relies heavily on the re-use and sharing of data. Biomedical data, however, is subject to strict data protection requirements. Due to the complexity of the data required and the scale of data use, obtaining informed consent is often infeasible. Other methods, such as anonymization or federation, in turn have their own limitations. Secure multi-party computation (SMPC) is a cryptographic technology for distributed calculations, which brings formally provable security and privacy guarantees and can be used to implement a wide-range of analytical approaches. As a relatively new technology, SMPC is still rarely used in real-world biomedical data sharing activities due to several barriers, including its technical complexity and lack of usability.

**Results:**

To overcome these barriers, we have developed the tool *EasySMPC,* which is implemented in Java as a cross-platform, stand-alone desktop application provided as open-source software*.* The tool makes use of the SMPC method Arithmetic Secret Sharing, which allows to securely sum up pre-defined sets of variables among different parties in two rounds of communication (input sharing and output reconstruction) and integrates this method into a graphical user interface. No additional software services need to be set up or configured, as EasySMPC uses the most widespread digital communication channel available: e-mails. No cryptographic keys need to be exchanged between the parties and e-mails are exchanged automatically by the software. To demonstrate the practicability of our solution, we evaluated its performance in a wide range of data sharing scenarios. The results of our evaluation show that our approach is scalable (summing up 10,000 variables between 20 parties takes less than 300 s) and that the number of participants is the essential factor.

**Conclusions:**

We have developed an easy-to-use “no-code solution” for performing secure joint calculations on biomedical data using SMPC protocols, which is suitable for use by scientists without IT expertise and which has no special infrastructure requirements. We believe that innovative approaches to data sharing with SMPC are needed to foster the translation of complex protocols into practice.

**Supplementary Information:**

The online version contains supplementary material available at 10.1186/s12859-022-05044-8.

## Background

### Introduction

Biomedical research is becoming increasingly data-driven [1]. To create the large datasets needed to answer precise scientific questions, data needs to be re-used for more than the initial purpose of collection and shared between different actors in the healthcare system and the research community [2–7]. As a consequence, “data sharing” is endorsed by various funding agencies (e.g., [8–10]) and increasingly implemented in practice [11, 12]. The term "data sharing" is used in a variety of ways. In this paper, we use it to refer to joint analyses of data stored at different institutions, which does not necessarily require the exchange of individual-level data. In research, data sharing can enable the generation of new knowledge (e.g., [13]) and also lead to higher citation rates [14, 15]. In addition to the increasing promotion of data sharing, there are also major hurdles to its adoption. Here, data protection and data privacy concerns are a central example (e.g., [7]). However, patients and the public have a positive attitude toward data sharing as long as their privacy is being protected [16–18].

Important laws protecting the privacy of patients and probands include the US Health Insurance Portability and Accountability Act (HIPAA) [19] and the EU General Data Protection Regulation (GDPR) [20]. Re-using or sharing data typically requires either (1) obtaining informed consent or (2) anonymizing the data [21]. However, on the one hand, obtaining consent is often infeasible, e.g., when data is analyzed in retrospect [22]. Anonymization, on the other hand, requires making inherent trade-offs between the degree of protection and the quality and hence utility of output data [23], often rendering individual-level data unsuited for answering medical research questions. As a result, a range of alternative approaches have been developed [24]. One example are distributed data sharing networks, in which no individual-level data, but aggregated results, are being shared amongst the partners to perform various types of joint analyses [25–27]. However, also this approach has limitations, for example when very small patient populations, e.g., with rare diseases, are to be studied, whose data cannot be aggregated [28].

Secure multi-party computation (SMPC) is an emerging cryptographic technology [29–31], which can be used to address the shortcomings of federated data networks. On an abstract level, SMPC protocols provide guarantees comparable to those of a trusted third party, with which the participating parties share their data with [32]. This trusted third party performs joint analyses and sends only the results back to the participants. The involved parties do not directly exchange data with each other and hence no information is being disclosed between them. SMPC can provide exactly the same guarantees by following specific cryptographic protocols that exchange encrypted data between the parties—without a trusted third party being involved. SMPC offers provable security guarantees and clearly stated assumptions. Especially for extremely sensitive information, including various types of biomedical data as targeted in this work, those strong guarantees provide a way to perform distributed analyses that otherwise could not be performed due to data protection challenges.

As a relatively new technology, SMPC has only been implemented for practical data sharing in the last few years [33–35] and it has been argued that this is the case in biomedical research as well [36, 37]. While some examples have been described in the literature, e.g., for survival analyses, genome-wide association studies [38–41], genomic diagnostics, detection of adverse drug events, or infection numbers during the COVID-19-epidemic [42] (see Section “Comparison with Prior Work”), these are mostly research prototypes or specific implementations of SMPC for specific analyses in the context of specific projects. There are several reasons for the slow adoption of SMPC technologies, amongst which are legal barriers, communication barriers, technical barriers and usability challenges (see “Limitations and future work” section).

### Challenges and objectives

In the work described in this paper, we addressed two important barriers—technical complexity and usability—to foster the adoption of SMPC technologies for biomedical data sharing:

1. Technical complexity: To enable distributed analyses of data across institutions, external queries against local IT solutions must be allowed and responses must be returned. This requires the installation of local services and an opening up of institutional firewalls. Both needs to be done with great care, which can lead to high efforts and potentially a reluctance to participate in data sharing networks.
2. Usability: SMPC protocols are typically implemented as command-line applications or provided as programming libraries (e.g., for statistical computing environments), thus addressing technical specialists, data scientists or other SMPC researchers. This makes it difficult for scientists involved in biomedical research projects, such as clinicians, to engage in SMPC-based data sharing.

We tackled these challenges by developing *EasySMPC*, which provides a “no-code solution” for securely performing joint calculations on distributed data using an intuitive graphical application. Moreover, no local services need to be installed and no permissive network configuration is necessary, as the application uses e-mails to exchange data between the participants while executing its protocol. To demonstrate the practicability of our solution, we evaluated its performance in a wide range of data sharing scenarios.

## Implementation

### Secure multi-party computation

SMPC describes a field of cryptographic techniques concerned with joint computations while maintaining confidentiality guarantees regarding the parties’ secret inputs. The field emerged in the 1980s with Andrew Yao’s publication of the “Garbled Circuits” protocol [43]. Another widely used SMPC method is the GMW-Protocol [44], which describes a way to securely compute a joint (Boolean) function on the secret inputs of $n$ parties. The underlying Boolean circuit uses only logical AND and XOR operations (that is, it states the function in algebraic normal form).

The GMW protocol can easily be extended to not only operate on Boolean circuits with logical values, but also on Arithmetic circuits with values of a finite ring. The idea of the secret sharing scheme is the same in both variants: generate shares (henceforth called “secret shares”) by mixing the secret value with randomness so that the combination of all shares results in the reconstructed secret. In the joint arithmetic computation, additions can be evaluated locally and multiplications are evaluated using interactive sub-protocols, such as the Gilboa-Multiplication for the two-party case [45].

This arithmetic extension of the GMW protocol, referred to as *Arithmetic Secret Sharing*, is the central method implemented in EasySMPC. For further information, we refer interested readers to Additional file 1 of this paper and to the literature (the book by Evans et al. provides a good starting point [46]).

### Design of EasySMPC

#### General approach

The general idea of *EasySMPC* is to provide a user-friendly tool for making SMPC-based data sharing available through an intuitive interface. EasySMPC uses Arithmetic Secret Sharing over the finite ring ${\mathbb{Z}}\left({2}^{127}-1\right)$, that is a ring of integers with ${2}^{127}-1$ elements. This assures, that for all practical values and number of parties the computation will not be restricted by the size of the finite field.^1^ As we only employ addition in this version, the protocol can be evaluated with two rounds of communication: first one round of sending/receiving shares for the values that are to be kept secret (e.g., case numbers of a rare disease in a hospital), hence revealing no information, and then a second round of sending/receiving shares for the intermediate results which can then be recombined to obtain the final result. As an inherent property of this family of secure protocols, this can be implemented without exchanging cryptographic keys in the classical sense during set up or prior to a computation, which is an additional factor contributing to the usability of the tool. Finally, we note that the scheme used by EasySMPC is a "full-threshold" protocol, meaning that it is robust against up to *n* − 1 corrupted parties, where *n* is the total number of participating parties, thus, providing a very high degree of protection.

From the user perspective, EasySMPC uses three concepts: (1) *Studies* are the overarching concept composed of participants, variables and protocol states; (2) *Participants* refer to different people or institutions, such as hospitals, who wish to engage in a common computation. Participants are identified by their name and e-mail address. Each study is initiated by exactly one study creator and involves two or more additional participants; (3) *Variables* refer to the data items that are independently summed up in one data sharing process and which are identified by unique names.

**Fig. 1:**
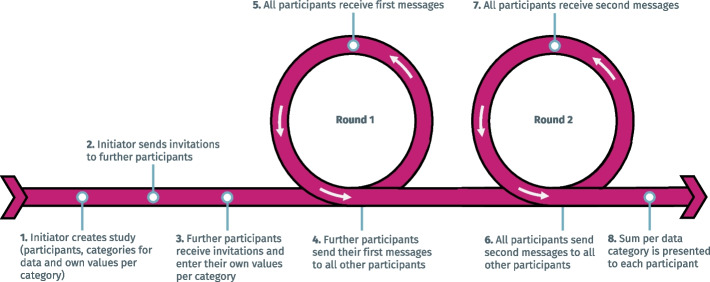
Overview of the steps in EasySMPC

Figure 1 provides an overview of the overall process implemented by EasySMPC and the different steps that users need go through when using the tool.

As depicted, the process consists of two rounds of data exchange: In the first round, meta-data and the shares for the participants’ secret values are exchanged. For this purpose, the study initiator creates the study, thereby providing a study name, a list of participants and their contact details as well as the list of (named) variables that will be summed up. The initiator also enters their own secret value for each variable, which will remain confidential. The sharable information is then sent to all other participants. Each participant receives their message, initializes the study and enters their own secret value for each variable, which will also remain confidential. Each participant (apart from the initiator) now sends a message to all other participants to distribute their respective secret share. Between communication rounds, each party calculates their new secret share locally by summing up the secret shares from round 1. In the second round, the same process is repeated, thereby exchanging the shares of the result. When a participant receives the final message, the result is reconstructed from the secret shares and the resulting sum for each variable across all participants is displayed. With *n* participants, each user sends and receives $2 \cdot \left(n-1\right)$ messages*.* That is, the number of messages for each participant grows linearly with the number of participants, implying that the *overall* number of messages sent during a calculation grows quadratically.

EasySMPC offers two ways of exchanging messages: (1) in the *semi-manual mode* the users exchange all messages by manually using their preferred e-mail client. The e-mails are, however, pre-generated by EasySMPC and can be imported automatically from the clipboard; (2) in the *automated mode* the participants receive and import the initial message manually. All further messages are exchanged automatically by an e-mail client built into the software.

#### Architecture and implementation of the software

**Fig. 2:**
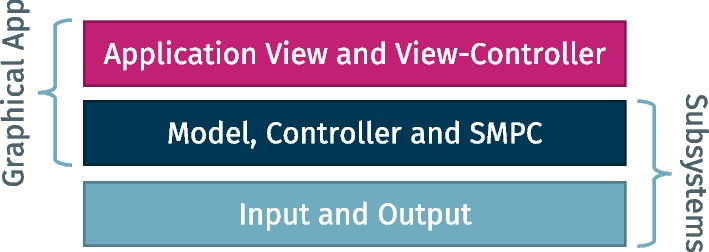
General architecture of EasySMPC

The architecture of EasySMPC follows the classic model-view-controller approach which is often used to implement applications with graphical user interfaces [47]. An overview of the most important modules is presented in Fig. 2.

EasySMPC is implemented in Java as a cross-platform, stand-alone application that was tested on Windows, MacOS and Linux. The graphical application is built on top of two subsystems, (1) one for cryptographic SMPC operations and (2) one for input- and output as well as data exchange with external applications and the other participants. The application itself consists of a module containing the different user-facing views and perspectives (described in more detail in the following section), as well as parts of the application controller, which is in charge of manipulating the model.

In detail, the three modules are designed as follows: (1) The *Application View and View-Controller* consists of eight different perspectives that reflect the process illustrated in Fig. 1 and guide users through its execution. For the perspectives, highly extendable components based on Java Swing were implemented. (2) The *Model, Controller and SMPC* module *is two-fold:* The module contains (a) the application model holding all data that is needed for executing the protocol and provides methods to safely switch between the states defined in the state machine (see below). Moreover, the module implements (b) the cryptographic Arithmetic Secret Sharing scheme presented in Additional file 1 of this paper. All interactions with this part of the subsystem are performed through the application model. (3) The *Input and Output (I/O) subsystem* provides functionalities for importing data from Excel and CSV files and for sending and receiving data by e-mail. A message can either be sent semi-manually by opening the user’s default e-mail client with all relevant fields (recipient, name of study etc.) pre-filled or in a fully automated manner by the I/O subsystem. In both cases the message itself is included in each mail as a Base64 encoded string. Each message contains all relevant metadata including the participants of the calculation, the name of all variables and the current state of the protocol execution, as well as a checksum to detect possible corruptions. Note, that a corrupted message may only lead to an erroneous result but cannot compromise input data privacy. A message can be received semi-manually by copying and pasting data into EasySMPC or be retrieved automatically by the I/O subsystem. In the first case, the application also monitors the user’s clipboard and automatically imports all EasySMPC-related messages that are contained in any text copied by the user. In the second case, a bus specifically developed for EasySMPC is used to exchange the data automatically between the different e-mail accounts.

**Fig. 3:**
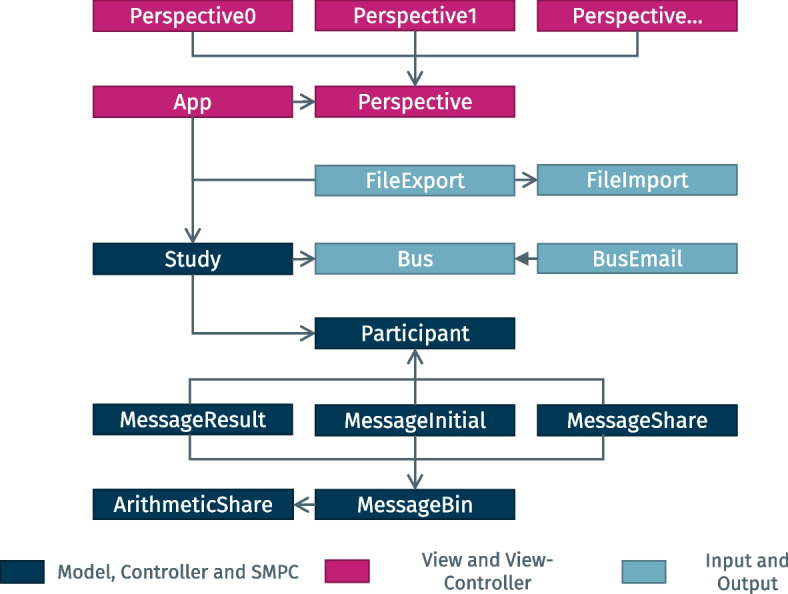
High-level class diagram

For the implementation, Java standard libraries as well as the libraries Jakarta Mail, Apache POI, Commons and Logging were used. Figure 3 displays a high-level class diagram of the software. The class *Study* is central to the execution of calculations through EasySMPC, as it implements the core algorithm. It makes use of further classes in the same module representing *Participants* as well as various types of messages and data used and exchanged. Data exchange is implemented through an abstract *Bus* system of which an implementation using e-mail is included. User interaction is controlled through the *App*, which contains the various perspectives described. It also acts as a mediator between the perspectives, the SMPC algorithm, data exchange and the tool’s data import and export capabilities.

**Fig. 4:**
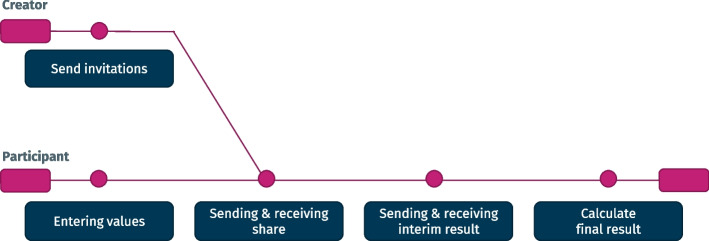
Application states

As mentioned, a finite state machine makes sure that the cryptographic protocol is followed as needed and that no invalid state transitions are being performed. The states and possible transitions are shown in Fig. 4. The state machine is also the reason why the application model, which handles the current state of the software, also contains parts of the controller. Given the asynchronous nature of data exchange, the API also allows saving the current state of the application at any time, not only after state transitions have been finalized.

## Results

### Overview of the software

**Fig. 5:**
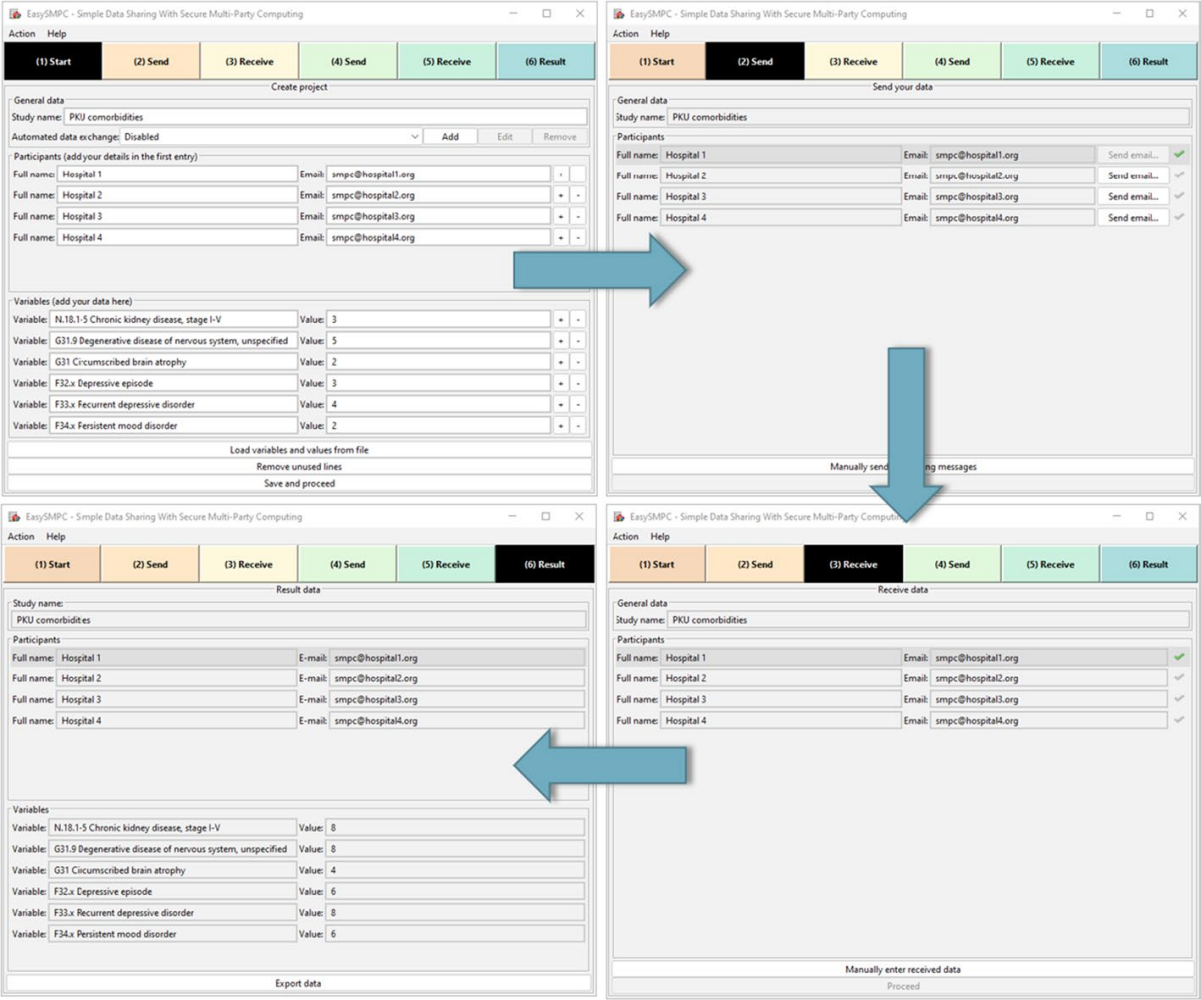
Perspectives of EasySMPC for (1) initializing a study, (2) sending messages, (3) receiving messages and (4) displaying the result

The different perspectives of EasySMPC are shown in Fig. 5. In the example, a common frequency distribution of co-morbidities of patients with Phenylketonuria (PKU), a congenital metabolic disease, is computed with four participating health care institutions. The figure shows the perspectives for (1) initializing a study, (2) sending messages, (3) receiving messages and (4) displaying the result. Similar perspectives that are used for the second round of the protocol have been omitted for brevity.

As can be seen, EasySMPC features a structured and intuitive design, in which data is displayed to the users in tabular form. A progress bar at the top of the application informs the user about the current step in the execution of the protocol. Important actions for the respective step are directly available in each perspective. Further operations, such as loading and saving a project, can be performed via the application menu.

### Performance evaluation

To evaluate the performance of EasySMPC we performed a wide range of experiments covering realistic application scenarios. Here we quickly provide an overview of results obtained using the default settings of EasySMPC. For a detailed description of the experimental setup and the results we refer to Additional file 2.

We varied two aspects: (1) the number of participants and (2) number of variables.

**Fig. 6:**
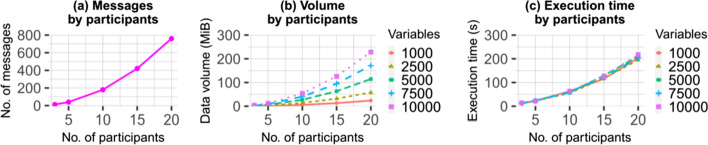
Experimental results obtained using the default settings

Figure 6a shows the total number of messages exchanged when processing the data of a varying number of participants while Fig. 6b, c show the total exchanged data volumes and execution times, which depend on the number of variables summed up as well as the number of participants.

In summary, our experiments confirm that the approach implemented by EasySMPC is feasible even in complex scenarios. The aggregation of 10,000 variables amongst 20 participants can be performed in less than five minutes.

The size of the messages exchanged by EasySMPC depends on the length of the names of the variables and the sizes of its values. The numbers obtained in our experiments show that, in a typical usage scenario, it can be expected that each variable-value-pair can be encoded in approximately 30 bytes (we used 10 random letters for each variable and values in the range of single-precision floating-point numbers). Many mail servers enforce a limit on the maximum size of messages that can be processed. Assuming a conservative limit of 10 Mbyte and based on the data obtained in our experiments this limit would be reached with about 340,000 variables. However, to support scenarios with even more variables, EasySMPC will split up larger messages into several smaller messages. The maximum message size is configurable in the software.

More details on the complexity of the algorithms involved is provided in “Computational complexity” section.

## Discussion

### Principal results

EasySMPC is a tool that allows summing up values of variables keeping the participants’ inputs confidential. To realize this, the software uses an established Arithmetic Secret Sharing protocol.

EasySMPC’s innovative aspects lie in the fact that it is very easy to roll out, as no additional effort for installing software services or configuring network interfaces is required and that it offers an intuitive user interface that addresses the needs of non-technical users, such as medical researchers. Through integration into the users’ desktop environments and existing e-mail infrastructures, the tool is able to leverage the most common communication channel that is likely to be readily available at sites wanting to engage in a common secure calculation. By using multiple rounds of calculations, several important statistical analyses can be realized (see next section). We have demonstrated its practicability by an extensive evaluation. EasySMPC is released as open-source software under a permissive license and its source code is available online [48].

### Supported data analyses

To make EasySMPC as easy to use as possible, the range of supported functionality has been kept to a minimum, focusing on the secure addition of a pre-defined set of variables. However, this basic functionality can be used to perform a range of more complex statistical analyses. For this purpose, different (derived) variables can be processed in multiple cycles, where each cycle is defined as one execution of EasySMPC, i.e., two rounds of sending and receiving messages. An overview of how the most fundamental statistical methods in biomedical research, as identified by Scotch et al. [49], can be implemented with EasySMPC is provided in Table 1.

**Table 1:** Example of common statistical methods that can be implemented with EasySMPC

Statistical method	Level of measurement	Input data^a^	Cycles with *EasySMPC*
Frequency distribution	Nominal	Local frequencies per class	1
Chi-square test	Nominal	Local frequencies per cell	1
Quartiles (median, interquartile range)	Ordinal	Local frequencies per class	1
Wilcoxon rank sum test	Ordinal	Local frequencies per class	1
Mean	Interval	Local sum and local count of values	1
Standard deviation (SD)	Interval	Data for mean and local deviation of mean	2
t-test/analysis of variance (ANOVA)^b^	Interval	Local sum, local count of values and local deviation of group mean	2
Correlation coefficient^c^	Interval	SD per variable, co-variance per variable	3

^a^All participants learn the global sum of the data entered locally. No participant learns local values of the other participants

^b^t-test is a special case of the analysis of variance with two groups

^c^Only possible if data for both variables to be correlated are available at the parties (horizontal data distribution)

The table shows that a range of analyses can be performed with one cycle in EasySMPC. Most of these analyses are suited for variables with a nominal level of measurement (indicating that the values have no natural order) and variables with an ordinal scale of measure (indicating that values have a natural order, but no relative distance between values can be expressed). Important examples include the computation of common frequency distributions (already mentioned above) and chi-square tests, where the cells of the relevant contingency table have to be defined a priori and cell counts can be summed up with EasySMPC to derive the final chi-square statistics. For ordinal data, quartiles can be derived from the common frequency distribution. Moreover, an inferential test of two independent distributions, the Wilcoxon rank sum test, can be performed using two common distributions computed with EasySMPC. For variables with an interval scale (indicating a natural order and a relative distance between values), further analyses are supported. For example, a common mean can be calculated by having each participant share a sum of a variable and the number of values, which can be divided with each other after computing common sums. Implementing further statistical analyses will require more than one cycle. For example, the standard deviation of a common distribution can be computed by calculating the mean in a first cycle. In a second cycle, each participant can calculate the variation of its data compared to the global mean. By using the variance computed in the second cycle and the total number of values calculated in the first cycle, the participants can further calculate the total standard deviation. In a third cycle, the total covariance can be computed to investigate a correlation for horizontally distributed data. Analogously, a t-test or analysis of variance can be performed by calculating the mean per group in a first cycle and the variance of local data in relationship to the global mean in a second cycle. When all those common sums are computed, the t-test and analysis of variance (ANOVA) statistics can be calculated.

We note that when an analysis is performed using more than one cycle, more data will be disclosed than when the complete process would have been performed using a tailored SMPC protocol. However, we would like to point out that, as already mentioned above, only aggregated and likely less sensitive data (cf. GDPR Recital 162 (5) [20]) is disclosed in the intermediate results. However, this needs to be carefully analyzed on a case-by-case basis before performing more complex analyses.

### Computational complexity

With its actual runtime being highly dependent on the employed (networking) hardware, the asymptotic complexities regarding runtime and space usage are important for evaluating the protocol. EasySMPC employs a SMPC protocol with a constant number of communication rounds and outside of those interactions only non-interactive, computationally inexpensive additions. This means that EasySMPC’s asymptotic runtime complexity is linear in the number of network interactions. The number of messages sent by each participant in a computation with $n$ participants is $2\cdot (n-1)$ (see also “Design of EasySMPC” section). This also means that it is unlikely that limits of typical mail servers regarding the number of messages that can be sent within a certain timeframe will be reached in calculations with a reasonable number of participants. The *overall* number of messages, which determines runtime performance, is $\mathcal{O}({n}^{2})$, which is executed in a parallel manner over $n$ concurrent processes (one executed by each participant).

Space complexity, again, is dependent on the number of messages. The messages contain the variable names and values, as well as a small overhead. Each individual message scales linearly in the number of variables. The overall space complexity of EasySMPC therefore is $\mathcal{O}(v{\cdot n}^{2})$ with $v$ being the number of variables, where each participant needs memory of $\mathcal{O}(v\cdot n)$.

Lastly, the consecutive execution of EasySMPC to create the more complex analyses listed in Table 1 (see “Supported data analyses” section) compose linearly, as all examples use the same number of participants and variables for each iteration. As the number of iterations is small in every given case, the incurred small factor can be omitted in an asymptotic complexity analysis.

### Comparison with prior work

A number of SMPC protocols and solutions have already been described in the literature that can be used in different areas of biomedical research. For example, Stammler et al. [41] and other authors [50–52] have investigated general secure record-linkage processes [53]. Moreover, El Emam et al. describe a protocol for the secure linkage of data for surveillance registries [54]. Several works describe the application of SMPC techniques for specific use cases in biomedical research. Examples include methods for conducting drug-target interaction assessments [55, 56], drug screening [57], genome-wide association studies [38, 39, 58–63] and genomic diagnostics [64]. Other works propose the application of SMPC techniques to realize specific statistical methods allowing biomedical data analyses, such as (1) the calculation of Kaplan–Meier estimators [65, 66], (2) linear [67] or (3) logistic [68–71] regression analyses and k-means clustering [72]. In addition, there are generic frameworks that can be used as a basis for implementing specific SMPC algorithms. Important examples include technical programming libraries and environments such as Sharemind MPC [73], FRESCO [74], ABY [75], MOTION [76] or MP-SPDZ [77] and generic data sharing infrastructures, such as MedCo [78] or FAMHE [79]. Tools that specifically target usability are also a hot topic in the biomedical field (see, e.g., [80, 81] for recent examples).

The papers cited in the first three areas describe complex algorithms which have been developed for a particular purpose. EasySMPC, on the other hand, follows a different strategy and supports a generic functionality optimized for usability by people that are not IT specialists. Moreover, we note that EasySMPC is not a research prototype but has been designed for real-world applications. The same is true for MedCo and FAMHE, which provide more comprehensive functionalities than EasySMPC. However, the efforts required to install, configure and maintain these solutions is relatively high, while EasySMPC was designed to be as easy as possible to install and use.

### Limitations and future work

The current restriction of EasySMPC to addition and subtraction is a major limitation of the software. While, as we have shown, this basic functionality can be used to implement a range of analyses, this can be cumbersome, as several independent rounds need to be performed. In future versions of the tool, we plan to add support for additional basic operations as well as more complex data analyses. On the user interface level, we plan to maintain EasySMPC’s usability by using a spreadsheet-like approach for entering data and displaying results.

In addition to the controlled experiments presented in this paper, we have also performed feasibility evaluations with EasySMPC in a real-world setting involving several hospitals from the German CORD project for research on rare diseases. While EasySMPC worked very well in all of those settings, the use of e-mail as a communication infrastructure resulted in some limitations. One example is that common mail servers may flag communication as spam if a very large number of messages is exchanged due to a large number of participants being involved. To also support such use cases, work is currently underway to extend the bus functionality of EasySMPC to other common communication technologies.

On the security and privacy-side, some trade-offs had to be made. First, the different parties are only authenticated via access to the e-mail accounts, meaning that a man in the middle attack could be performed and the integrity of the calculation cannot be guaranteed. However, this does not affect the confidentiality of the data entered by the participants, since the employed protocol is proven to be secure [44]. Thus, in the worst case, an attacker might maliciously change the calculated results, but is never able to obtain the input data of other participants. Moreover, like many other SMPC solutions [34], EasySMPC provides a safe setting for processing data but does not necessarily guarantee that the output data is also protected (see also “Supported data analyses” section). In future work, we plan to address these issues by integrating more comprehensive authentication mechanisms and methods for providing safe outputs, such as Differential Privacy [82].

Finally, there are a few general barriers to the further adoption of SMPC methods that are not specific to EasySMPC. For example, Tõldsepp et al. [83] identified the following important challenges that also apply to our software: (1) legal frameworks often do not consider SMPC, methods which in turn leads to legal uncertainties (see also [37]), (2) it can be challenging to explain and communicate the properties of SMPC to relevant stakeholders (e.g., Institutional Review Boards (IRBs) or ethics committees; see also [37, 46, 84]), (3) users may misuse SMPC technologies leading to additional risks in the *honest but curious* attacker model typically assumed (see also [85]) and (4) data analysts might find it difficult to analyze data they cannot access directly (see also [46, 86]). By developing EasySMPC which makes such technologies available to a broader audience and more use cases, we hope to be able to contribute to overcoming these barriers.

## Conclusions

In this paper we have presented EasySMPC, a user-friendly graphical application supporting the secure analysis of distributed data across multiple institutions without requiring IT expertise. Although SMPC methods are considered a break-through technology for data-driven medical research, they are not in widespread use to date and implementing them can be associated with major hurdles. We believe that innovative no-code approaches to secure data sharing, as the one presented in this paper, can foster the translation of more complex protocols into practice.

### Availability and requirements

Project name: EasySMPC. Project home page: https://github.com/prasser/easy-smpc. Operating system(s): Platform independent. Programming language: Java. Other requirements: Java 14 or higher. License: Apache 2.0. Any restrictions to use by non-academics: none.

## Supplementary Information

**Additional file 1.** Microsoft Word format describes the employed SMPC method in detail.

**Additional file 2.** Microsoft Word format contains the detailed results of the performance evaluation.

## Data Availability

The performance evaluation dataset generated and analyzed during the current study is available in the GitHub repository of the performance evaluation, https://github.com/fnwirth/easy-smpc-performance-evaluation.

## Abbreviations

ANOVA: Analysis of variance
GDPR: General data protection regulation
HIPAA: Health Insurance Portability and Accountability Act
I/O: Input and output
IRB: Institutional Review Board
OT: Oblivious transfer
PKU: Phenylketonuria
SD: Standard deviation
SMPC: Secure multi-party computation
XOR: Exclusively-OR

## Footnotes

1 We note that EasySMPC nevertheless supports the summation of decimal numbers by using a fixed-point representation.
