# Supplementary material for: EasySMPC: a simple but powerful no-code tool for practical secure multiparty computation
Source: BMC Bioinformatics. 2022 Dec 9;23:531. doi: 10.1186/s12859-022-05044-8 (PMC9733077; doi:10.1186/s12859-022-05044-8)
Supplement: Supplementary file 1 — Additional file 1. Microsoft Word format describes the employed SMPC method in detail. [file 12859_2022_5044_MOESM1_ESM.pdf]

Additional File 1: Secure Multi-Party Computation (SMPC)

SMPC describes a collection of cryptographic techniques concerned with computations among several parties while maintaining privacy guarantees regarding the parties' secret inputs. The field emerged in the 1980s with Andrew Yao's publication of the "Garbled Circuits" protocol [1]. The fundamental insight, that by representing a function as a discrete circuit, either a Boolean circuit, operating on logical values, or an arithmetic circuit operating on elements of a finite ring, every computation that can be executed by a trusted third party can also be securely performed using a cryptographic protocol started this new research field.

Albeit theoretically feasible, SMPC was missing practical implementation until the early 2000s. Due to vast improvements in both the performance of computing and network equipment and optimizations and novel approaches in SMPC protocols, the techniques became practically feasible in recent years. Today, the performance penalty by employing SMPC techniques is still high – sometimes multiple magnitudes increase in execution times compared to non-secure evaluation, nevertheless many practical problems can be solved using a multitude of SMPC frameworks and techniques.

In the following two sections we will briefly describe the protocols which we used in the work described in this article.

1.1 The GMW Protocol

The GMW-Protocol [2], named after the inventors Goldreich, Miciali and Widgerson, operates on a Boolean circuit representation of a function that is to be evaluated. This representation uses only logical AND and XOR operations (that is, it states the function in algebraic normal form). Nearly all functions can be stated in this form, when some boundary conditions are met, e.g., that loops are bound and can be unrolled. More technically: Every polynomial-time function can be represented in a Boolean circuit of polynomial size. Following the terminology of electrical engineering, every operation in the circuit is called a *gate* and those gates are connected via *wires* that each hold one bit.

The privacy of each party's input is guaranteed by utilizing a threshold-cryptographic secret sharing scheme, that means the input is "broken up" into multiple parts, each on its own not containing any information, which allow reconstruction of the secret value only if all parts are combined. The secret value cannot be reconstructed, if even a single part, called a "share", is missing. This statement holds for adversaries with unlimited computational power and time.

The GMW protocol describes a way to securely compute a joint (Boolean) function on the secret inputs of n parties or institutions. The idea of GMW's information-theoretically secure secret sharing is to "break up" the secret input bit of each party into n shares by sampling $n - 1$ random bits and generating the last share by XORing all previous shares with each other and the secret input bit. This construction is, given a true random number generator, information theoretically secure, which means, that even an adversary with unlimited computational power and time cannot infer any information regarding the secret bit without access to *all* n shares of a value.

After the secret shares are generated, each party receives one share of every party's' input. No party has enough shares to reconstruct any secret value, but the computations can be performed on those shares. In the case of XOR-gates the operation can be performed locally, that is: The result of XORing one share of each input value of the gate is exactly the secret share of the calculation's result. AND-gates, however, require an

interactive sub-protocol involving communication between all parties using Oblivious Transfer (OT) [3–5].

After evaluating the Boolean circuit both parties recombine the resulting shares to reveal the clear text value of the output.

1.2 Arithmetic Secret Sharing

The GMW protocol can easily be extended to not only operate on Boolean circuits with logical values, but on arithmetic circuits (consisting of additions and multiplications, instead of the Boolean operations AND and XOR) with values of a finite ring, as well. The idea of the secret sharing scheme remains the same: generate shares by mixing the secret value with randomness so that the combination of all shares results in the reconstructed secret. The construction of the secret shares is analogous to the Boolean case, using modular arithmetic on the ring $\mathbb{Z}(p)$: to generate n shares (to be distributed to the n parties) sample *n-1* random elements of the finite ring, or formally $s_i \leftarrow_{\$} \{v | v \in \mathbb{Z}(p)\}, i = 1, \dots, n-1$. The last share, the nth, is generated by subtracting the previously sampled random values from the secret value, modulo the ring size to "stay inside" of the ring: $s_n = v - \sum_{i=1}^{n-1} s_i \bmod p$.

The evaluation of the arithmetic circuit is an extension of the GMW protocol as well: additions can be evaluated locally and multiplications are evaluated using sub-protocols, like for the two-party case the Gilboa-Multiplication [6]. Without loss of generality, we can easily show the "locality" of the addition-operation, where s_i^p denotes the ith share of the secret value v^p of party p by writing out each definition and operation as illustrated in Table 1:

Table 1: Illustration of arithmetic Secret Sharing

	**Party 1**	**Party 2**
**Holds**	$s_2^1 = v^1 - s_1^1 \bmod p$	$s_1^1 = random$
	$s_1^2 = random$	$s_2^2 = v^2 - s_1^2 \bmod p$
$\boldsymbol{v^1 + v^2}$	$\boldsymbol{r^1 = s_2^1 + s_1^2 \bmod p}$	$\boldsymbol{r^2 = s_1^1 + s_2^2 \bmod p}$
	$\boldsymbol{= v^1 - s_1^1 + s_1^2 \bmod p}$	$\boldsymbol{= s_1^1 + v^2 - s_1^2 \bmod p}$
**Reconstruction (both parties)**	$\boldsymbol{r^1 + r^2 \bmod p = v^1 - s_1^1 + s_1^2 + s_1^1 + v^2 - s_1^2 \bmod p}$	
	$\boldsymbol{= v^1 + v^2 \bmod p}$	

1.3 References

[1] Yao AC-C. How to generate and exchange secrets. SFCS '86: Proceedings of the 27th Annual Symposium on Foundations of Computer Science; 27 - 29 October 1986. Washington: IEEE Computer Society; 1986, p. 162–7. https://doi.org/10.1109/SFCS.1986.

[2] Micali S, Goldreich O, Wigderson A. How to play any mental game. In: Aho A, editor. STOC '87: Proceedings of the Nineteenth ACM Symposium on Theory of Computing; 25 - 27 May 1987; New York. New York: Association for Computing Machinery; 1987, p. 218–29. https://doi.org/10.1145/28395.28420.

[3] Asharov G, Lindell Y, Schneider T, Zohner M. More efficient oblivious transfer and extensions for faster secure computation. In: Sadeghi, Ahmad-Reza, editor. Proceedings of the 2013 ACM SIGSAC conference on Computer & communications security; 4 - 8 November 2013; Berlin. New York: Association for Computing Machinery; 2013, p. 535–48. doi: 10.1145/2508859.2516738.
[4] Asharov G, Lindell Y, Schneider T, Zohner M. More Efficient Oblivious Transfer Extensions. J Cryptol. 2017;30:805–58. doi:10.1007/s00145-016-9236-6.
[5] Ishai Y, Kilian J, Nissim K, Petrank E. Extending oblivious transfers efficiently. In: Boneh D, editor. CRYPTO 2003: 23rd Annual International Cryptology Conference; August 17 - 21, 2003; Santa Barbara. Berlin: Springer; 2003, p. 145–61. doi:10.1007/978-3-540-45146-4_9.
[6] Gilboa N. Two Party RSA Key Generation. In: Wiener M, editor. CRYPTO 99: 19th Annual International Cryptology Conference; 15 – 19 August 1999; Santa Barbara. Berlin, Heidelberg: Springer; 1999, p. 116–29. https://doi.org/10.1007/3-540-48405-1_8.
