# Supplementary material for: EasySMPC: a simple but powerful no-code tool for practical secure multiparty computation
Source: BMC Bioinformatics. 2022 Dec 9;23:531. doi: 10.1186/s12859-022-05044-8 (PMC9733077; doi:10.1186/s12859-022-05044-8)
Supplement: Supplementary file 2 — Additional file 2. Microsoft Word format contains the detailed results of the performance evaluation. [file 12859_2022_5044_MOESM2_ESM.pdf]

Additional File 2: Performance evaluation

1.1 Performance evaluation setup

To evaluate the performance of EasySMPC we performed a wide range of experiments covering realistic application scenarios. We varied two technical factors as well as two user-specific factors. The two technical factors were:

(1) *Polling frequency:* The time interval at which EasySMPC automatically checks for incoming messages (settings used: 1, 5, 10, 15 and 20 seconds).

(2) *Network latency:* The delays in communication over the network, which typically increases with distance (settings used: 30 milliseconds to simulate national data sharing, 100 milliseconds to simulate international data sharing).

By default, the polling frequency is set to 5 seconds and the network latency is set to 30 milliseconds.

There was no need to limit the bandwidth of the connections used by the participants, as the number of messages exchanged is the most important factor and the overall data volume exchanged by each participant is quite small (not more than about 10 MB in the most complex setting).

The two user-specific factors were:

(1) *Number of participants:* The number of institutions involved in the computation (settings used: 3, 5, 10 and 20).

(2) *Number of variables:* The number of variables summed up in a calculation (settings used: 1000, 2500, 5000 and 10000).

To allow experimenting with a wide range of settings, a testbed was created consisting of a single machine with 128 1.8 GHz CPUs having 32 cores each and 512 GB of RAM running CentOS 8.4. The machine ran a dockerized evaluation setup which was equipped with a mail server (iRedMail); the tool 'tc'[1] was used to introduce network latency. EasySMPC was executed on an Oracle JRE (version: 14.0.1). The code and the docker files used in the evaluation are available online [1].

For each possible combination of all technical and user-specific factors we performed 15 experiments with EasySMPC's command-line mode. The collected outcome variables for each experiment where (1) the number of messages exchanged, (2) the total data volume transferred and (3) the time needed to complete the calculation. We report average execution times in the "Results" section.

1.2 Results with a network latency of 30 milliseconds

The experiments have shown that the higher latency of 100 milliseconds only leads to a doubling of the execution times on average, but has no influence on the observed underlying patterns and relationships. Hence, we focus on the results obtained with 30 milliseconds latency in this section first and report the numbers for higher latencies in the next section.

Figure 1 provides insights into the number of messages and data volumes exchanged in the experiments.

---

[1] https://man7.org/linux/man-pages/man8/tc.8.html

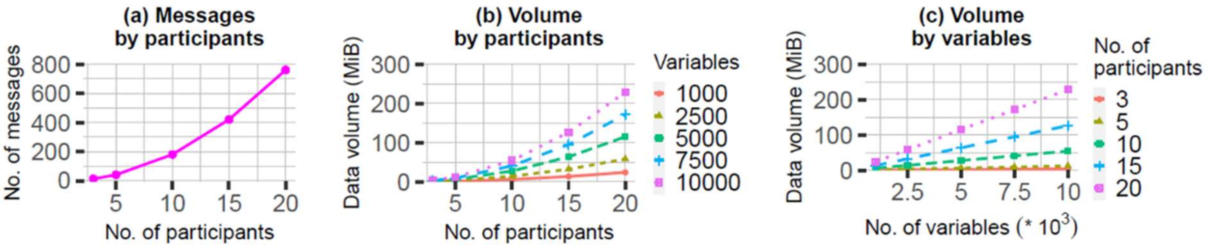

Figure 1: Number of messages and total data volume exchanged.

Figure 1a illustrates the quadratic growth of the number of messages exchanged when the number of participants increases (see also Section “General Approach” of the main manuscript). As expected, Figure 1b shows that the total data volume exchanged also increases quadratically with an increasing number of participants while increasing the number of variables adds a multiplicative factor. This is also illustrated by Figure 1c which shows a linear increase of the overall data volume exchanged with an increasing number of variables.

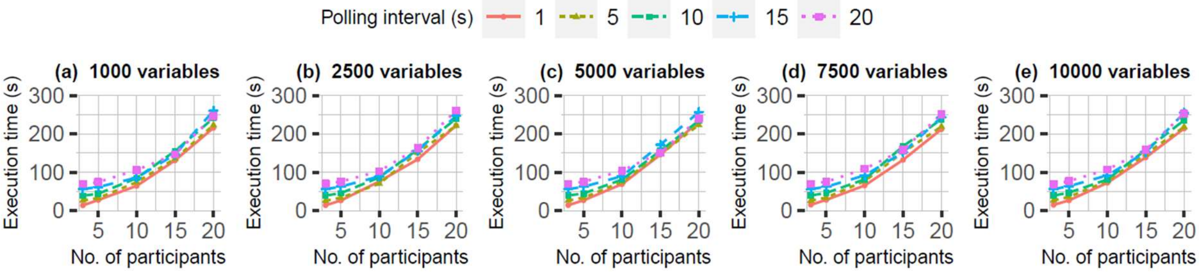

Figure 2: Execution times for increasing numbers of participants and variables as well as different polling frequencies.

Figure 2 shows execution times for different numbers of variables summed up amongst different numbers of participants using different polling frequencies. As can be seen, analogously to the relationship between the number of participants and the number of messages exchanged, also the execution times increase quadratically with the number of participants. As mentioned above, increasing the number of variables linearly increases the exchanged data volume. However, as can be seen from this figure, this only has a negligible impact on overall execution times. On the contrary, increasing the polling frequency, i.e., the interval in which the mailboxes are screened automatically, results in a linear increase of execution times with a factor inversely proportional to the number of participants.

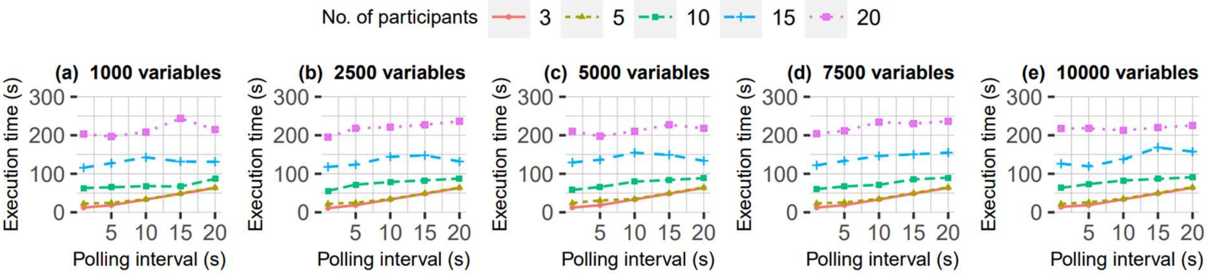

Figure 3: Details on the relationship between execution times and polling frequency.

Figure 3 presents a different view to better visualize the relationship between execution time and polling frequency. As can be seen, this view confirms that the polling frequency has a linear effect on execution times, which decreases with an increasing number of participants.

In summary, our experiments confirm that the approach implemented by EasySMPC is feasible even in complex scenarios. The aggregation of 10000 variables amongst 20 participants can be performed in less than five minutes.

1.3 Results with a network latency of 100 milliseconds

In this section, we report on the results of the performance evaluation when using a network latency of 100 milliseconds (ms). The trends displayed are the same as for a network latency of 30 ms, however, the total execution time was slower by a factor 2.1 on average.

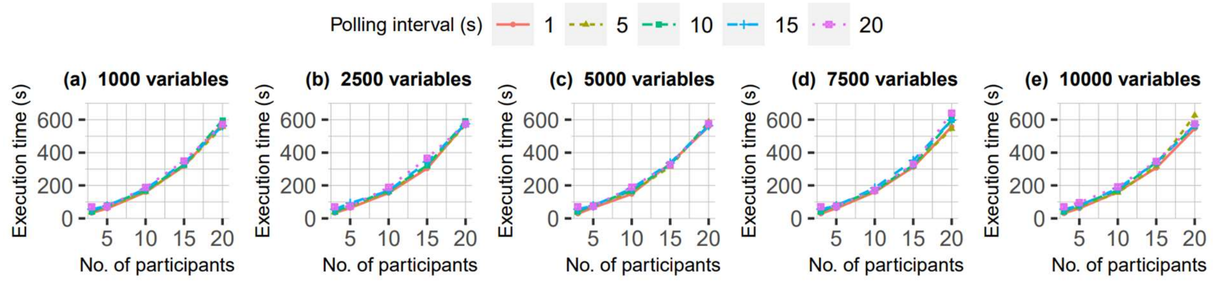

Figure 4: Execution times for increasing numbers of participants and variables as well as different polling frequencies.

Figure 4 shows the execution times measured for different numbers of variables summed up amongst different numbers of participants using different polling frequencies. As with a latency of 100 ms, the execution times increase quadratically with the number of participants. Furthermore, also in this scenario, increasing the number of variables had only a negligible impact on execution times.

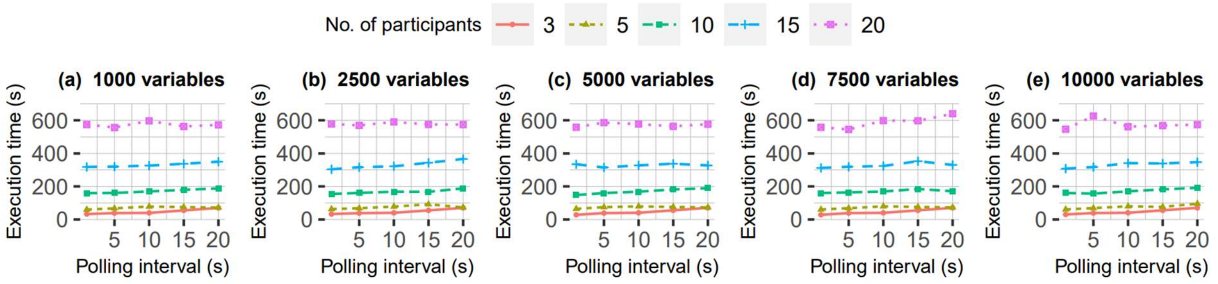

Figure 5: Details on the relationship between execution times and polling frequency.

Figure 5 presents the relationship between execution time and polling frequency for a 100ms network latency. As can be seen, also this scenario confirms that the polling

frequency has a linear effect on execution times, which decreases with an increasing number of participants.

1.4 References

1. Wirth FN, Kussel T, Müller A, Hamacher K, Prasser F. EasySMPC performance evaluation. https://github.com/fnwirth/easy-smpc-performance-evaluation. Accessed May 17 2022.
